# The role of *PPP2R1A* variants in gynecologic malignancies: a paradigm shift in immuno-oncology

**DOI:** 10.1186/s12967-026-08181-3

**Published:** 2026-04-30

**Authors:** Junying Chen, Yudi Tan, Yuying Wei, Yuxuan Jin, Jingwen Huang

**Affiliations:** Department of Obstetrics and Gynecology, Guangxi Zhuang Autonomous Region, First Affiliated Hospital of Guangxi Medical University, Shuangyong Road, Qingxiu District, Nanning City, People’s Republic of China

**Keywords:** *PPP2R1A*, Gynecologic cancer, Immuno-oncology, cGAS-STING pathway, Predictive biomarker

## Abstract

**Background:**

Protein Phosphatase 2 A (PP2A), a key tumor suppressor, is frequently inactivated in cancer through mutations in its scaffold-encoding gene, *PPP2R1A*. This is particularly common in aggressive gynecologic malignancies, specifically uterine serous carcinoma (USC) and ovarian clear cell carcinoma (OCCC). A compelling paradox has emerged: while these mutations are potent oncogenic drivers, they are also powerful predictive biomarkers for an exceptional response to immune checkpoint inhibitor (ICI) therapy.

**Main body:**

This study provides a comprehensive narrative review of the literature to dissect the dual role of *PPP2R1A* in gynecologic cancers. This review elucidates the molecular pathogenesis and immune activation mechanisms of *PPP2R1A* mutations, while also outlining emerging therapeutic strategies targeting this pathway.

**Conclusions:**

The dual function of *PPP2R1A* mutations represents a paradigm shift in the field. Understanding this pathway provides crucial new insights that are poised to advance the future of precision immuno-oncology for patients with gynecologic cancers.

## The *PPP2R1A*/PP2A axis: molecular architecture and tumor suppressor function

### The PP2A holoenzyme: a master serine/threonine phosphatase

The PP2A holoenzyme is a heterotrimeric protein complex, with its core structure comprising a scaffold A subunit, a catalytic C subunit, and a variable regulatory B subunit [1]. Together with Protein Phosphatase 1 (PP1), PP2A accounts for over 90% of all serine/threonine phosphatase activity within the cell, highlighting its central role in maintaining cellular homeostasis [2]. The immense functional diversity of this enzyme family stems from its subunit complexity. The human genome encodes two isoforms for both the A and C subunits (α and β, respectively) and over 15 distinct B subunits, which are categorized into four families: B (B55), B’ (B56), B’’, and B’’‘ [3]. This combinatorial diversity allows for the formation of over 90 functionally distinct PP2A holoenzyme complexes. The specific B subunit dictates the enzyme’s substrate specificity, subcellular localization, and regulatory activity, enabling its precise involvement in a vast array of cellular processes [4, 5].

### *PPP2R1A*: The critical scaffold subunit and its role in holoenzyme integrity

The *PPP2R1A* gene encodes the Aα subunit, which serves as the structural backbone of the PP2A holoenzyme. It functions as a “molecular scaffold,” coordinating the proper assembly of the catalytic C and regulatory B subunits, a prerequisite for forming a functional holoenzyme [6]. In most cell types, Aα is the most abundantly expressed A-subunit isoform [7]. Its paralog, PPP2R1B, encodes the Aβ isoform; although their protein sequences are highly homologous, these two isoforms are not functionally redundant and possess distinct biological roles [8]. Beyond forming the basic ABC trimer, the Aα subunit also participates in larger supramolecular structures, such as the STRIPAK complex that regulates the Hippo and MAPK signaling pathways, and the INTAC complex involved in transcriptional elongation, further underscoring its multifaceted regulatory roles in cellular signaling networks [9].

### Mechanisms of tumor suppression: a gatekeeper for key oncogenic pathways

PP2A exerts its potent tumor-suppressive function by directly dephosphorylating and inactivating a range of critical oncoproteins and signaling pathways [10, 11].

PI3K/AKT/mTOR Pathway: This is a central pathway regulating cell growth, proliferation, and survival. PP2A dephosphorylates the Ser-473 site of the AKT protein, preventing its full activation and thereby inhibiting downstream mTOR signaling [12]. Studies have shown that *PPP2R1A* haploinsufficiency can induce cellular transformation by activating the PI3K/AKT pathway [13].

MAPK Pathway: PP2A can dephosphorylate and inactivate key kinases in the MAPK pathway, such as MEK and ERK, thereby controlling cell proliferation [14]. Notably, certain *PPP2R1A* mutations (e.g., R183W) can paradoxically enhance oncogenic RAS-MAPK signaling and lead to resistance to MEK inhibitors [15].

Wnt Pathway: PP2A is also involved in regulating the Wnt signaling pathway, which is crucial in both embryonic development and tumorigenesis [16]. This function, as will be discussed later, exhibits a complex and paradoxical nature.

Other Key Substrates: The regulatory network of PP2A extends to other core oncoproteins, including c-Myc and β-catenin, further demonstrating its broad role as a tumor suppressor [17, 18].

**Fig. 1:**
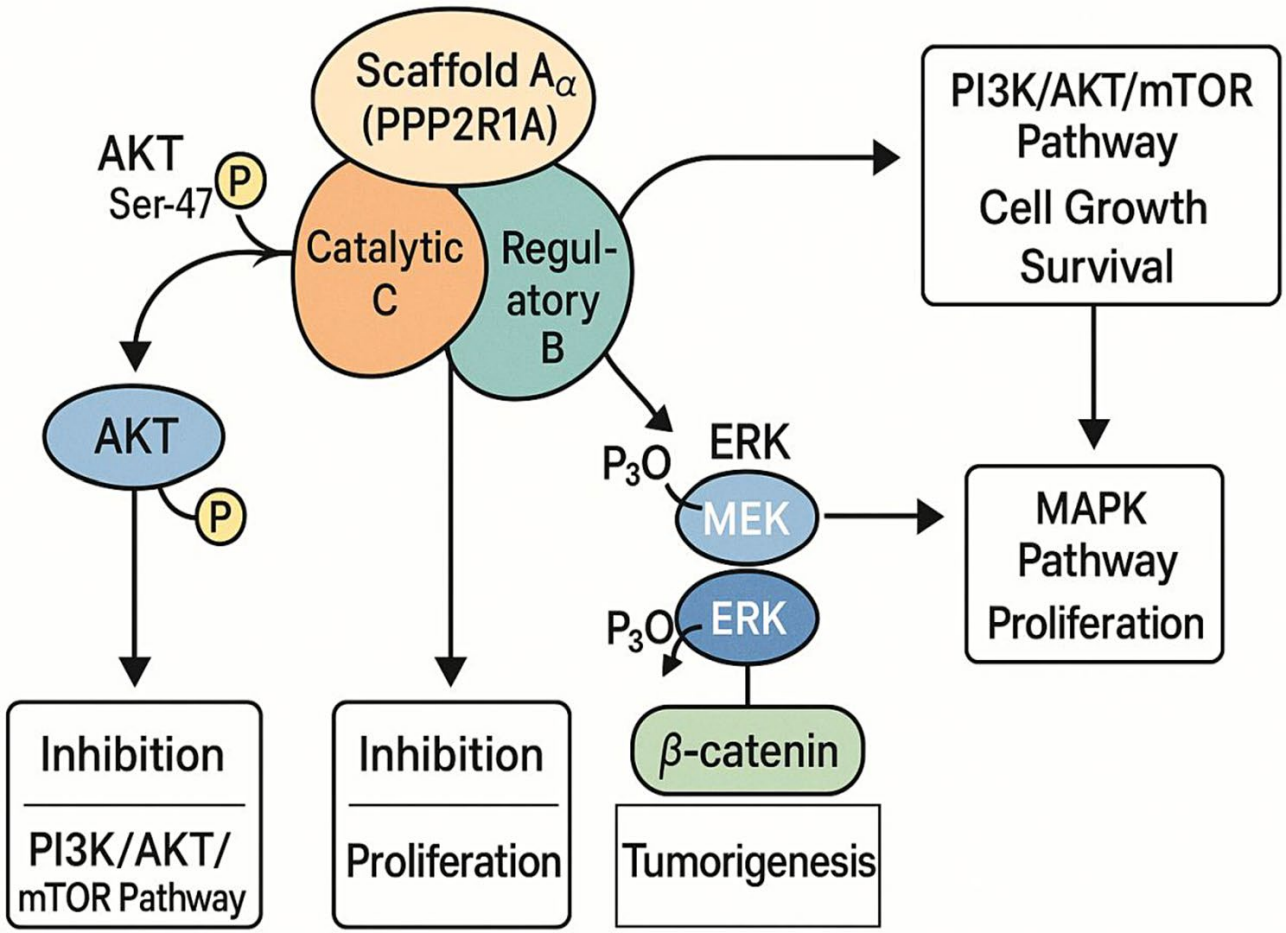
Canonical tumor-suppressive function of the wild-type PP2A holoenzyme. The PP2A holoenzyme, composed of the scaffold Aα subunit (encoded by *PPP2R1A*), catalytic C subunit, and regulatory B subunit, dephosphorylates multiple oncogenic kinases. PP2A removes the activating phosphate group from AKT at Ser-473, thereby inhibiting the PI3K/AKT/mTOR pathway and suppressing cell growth. Similarly, dephosphorylation of MEK and ERK attenuates MAPK signaling and reduces proliferation. PP2A also regulates β-catenin within the Wnt pathway, preventing tumorigenesis. Collectively, these actions underscore PP2A’s role as a master tumor suppressor

**Fig. 2:**
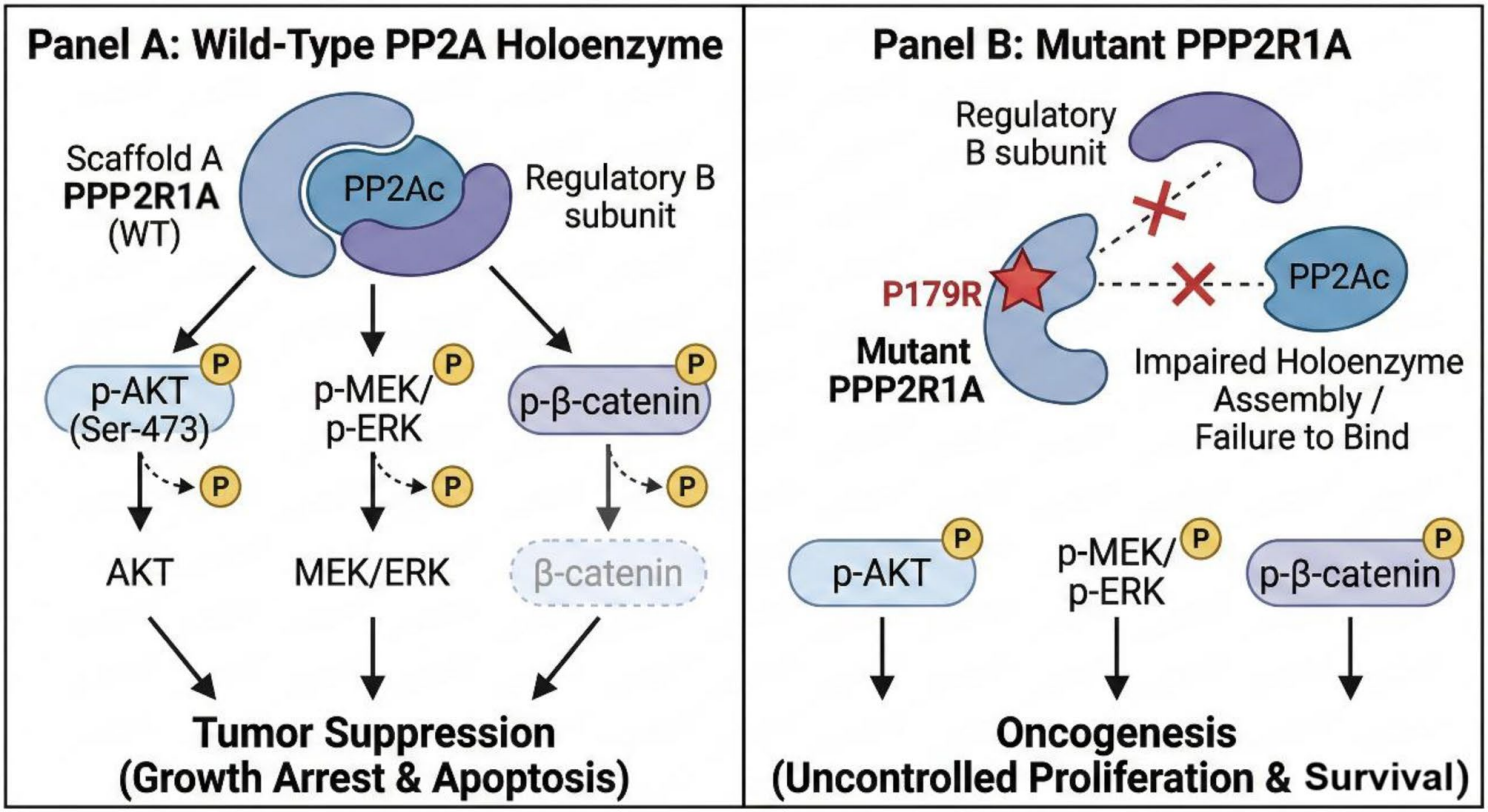
Structural disruption of the PP2A holoenzyme by PPP2R1A mutations and subsequent activation of oncogenic signaling. (**A**) In the wild-type state, the PPP2R1A scaffold subunit successfully coordinates the assembly of the catalytic (PP2Ac) and regulatory (**B**) subunits to form a functional PP2A holoenzyme. This intact complex exerts its tumor-suppressive function by directly dephosphorylating and inactivating critical oncogenic targets, including AKT (at Ser-473), MEK/ERK, and β-catenin, thereby restraining cellular proliferation. (**B**) The highly recurrent P179R mutation alters the structural conformation of the PPP2R1A scaffold. This mutation severely impairs the binding affinity for both the catalytic C and regulatory B subunits, leading to a failure in holoenzyme assembly. Consequently, PP2A-mediated dephosphorylation is abrogated, resulting in the sustained hyperactivation of the PI3K/AKT/mTOR, MAPK, and Wnt signaling pathways, which drive uncontrolled tumor growth and survival

The structural integrity of *PPP2R1A* is the cornerstone of PP2A’s tumor-suppressive function. Because the Aα subunit serves as the platform for assembling the catalytic C subunit and the substrate-determining B subunits, mutations in this single scaffold gene can trigger a catastrophic systemic failure [19]. These mutations disrupt the binding of the A subunit to either the B or C subunits, preventing the cell from correctly assembling a diverse array of tumor-suppressive PP2A holoenzymes. Consequently, multiple parallel and critical oncogenic pathways (e.g., AKT, MAPK, Wnt) become simultaneously dysregulated. For a cancer cell, achieving widespread signaling disruption through a “master switch” mutation in *PPP2R1A* is a far more efficient oncogenic event than accumulating individual mutations in components of each separate pathway. This explains why such mutations possess such potent oncogenic potential and are frequently selected for in highly aggressive cancers (Figs. 1 and 2).

## Clinical and pathological features of *PPP2R1A* mutations in gynecologic cancers

### Endometrial cancer (EC): a high-frequency and histotype-specific mutational hotspot

Among gynecologic tumors, endometrial cancer is a “hotspot” for *PPP2R1A* mutations, with a distribution that shows remarkable histotype specificity. In high-grade, Type II endometrial cancers, such as uterine serous carcinoma (USC) and uterine carcinosarcoma (UCS), the mutation frequency of *PPP2R1A* is exceptionally high, reaching up to 40% [20]. This stands in stark contrast to low-grade, Type I endometrioid endometrial cancer (EEC), where the mutation rate is very low, typically between 0% and 7% [21]. In Type I EC, PP2A function is more commonly inactivated through indirect mechanisms, such as the overexpression of its endogenous inhibitory proteins CIP2A and PME-1 [22].

**Fig. 3:**
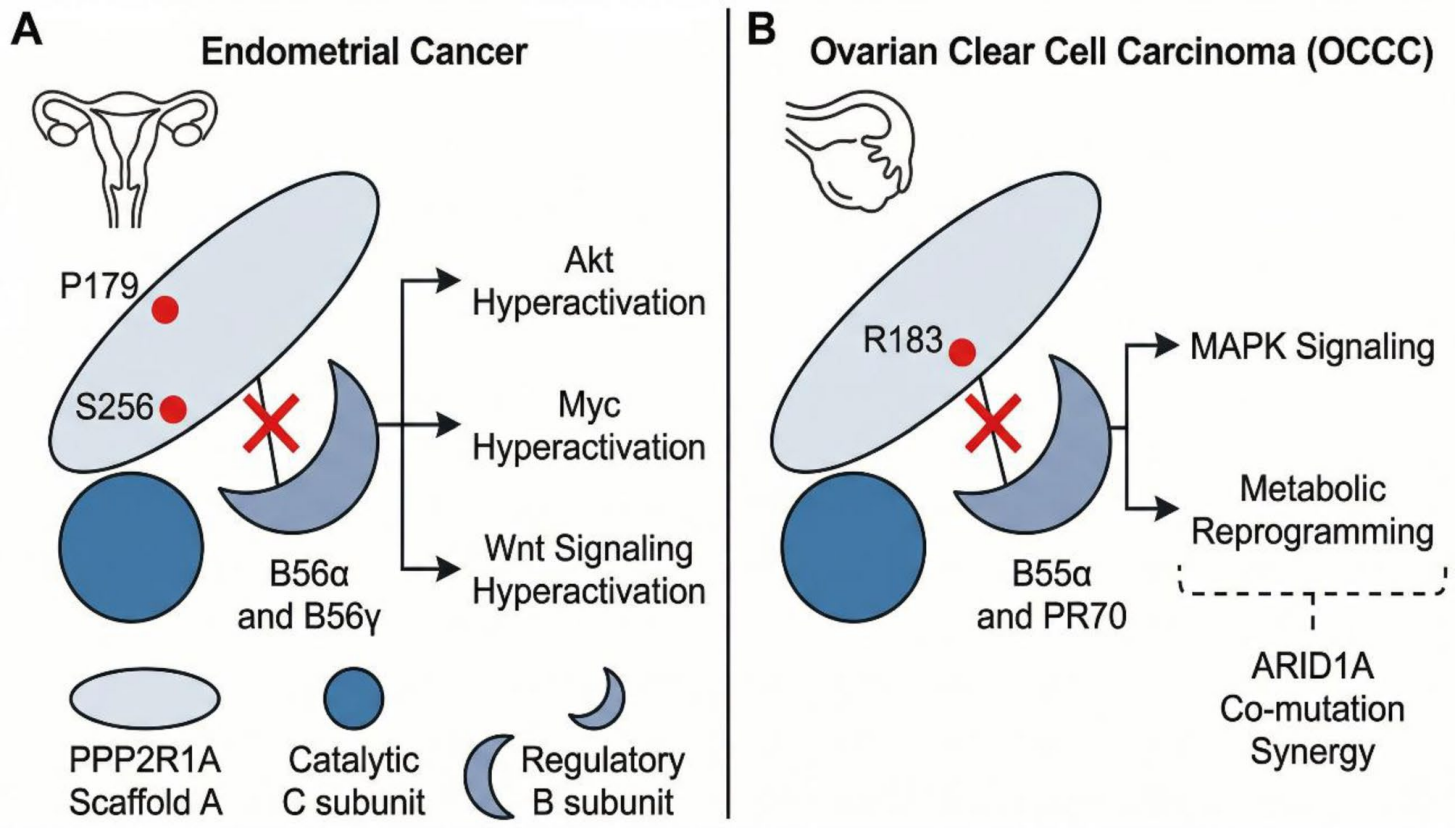
Modular schematic of tissue-specific PPP2R1A mutational hotspots and interactome disruption in gynecologic malignancies. Panel A illustrates the Endometrial Cancer (EC) context, where hotspot mutations cluster at codons P179 and S256 of the PPP2R1A scaffold. These structural alterations specifically impair the assembly of PP2A holoenzymes involving endometrium-enriched B subunits, including B56α and B56γ. This binding failure leads to the hyperactivation of critical oncogenic substrates, including Akt, Myc, and Wnt signaling. Panel B depicts the Ovarian Clear Cell Carcinoma (OCCC) context, characterized by the predominant R183 mutation. This mutation disrupts the recruitment of ovary-specific B subunits, including B55α and PR70, resulting in diminished regulatory control over the catalytic C-subunit. These molecular events drive sustained MAPK signaling and metabolic reprogramming, while exhibiting synergistic effects when co-occurring with ARID1A mutations

Even more striking is the unique “hotspot” distribution of *PPP2R1A* mutations in USC/UCS, which are almost exclusively clustered at codons P179 and S256. This high degree of tissue specificity remains a largely unsolved puzzle in current research [19]. Functionally, these hotspot mutations impair the binding of the A subunit to the B and C subunits, thereby diminishing the phosphatase’s activity [19, 23]. Some mutations also exert their effects through a dominant-negative mechanism, where the mutant protein acquires a gain-of-function ability to bind more strongly to the PP2A inhibitor TIPRL1, thus more effectively inhibiting wild-type PP2A function [24, 25]. Clinically, *PPP2R1A* mutations are highly correlated with another hallmark of Type II EC—*TP53* mutations. Multiple studies have associated *PPP2R1A* mutations with advanced-stage disease and poorer survival, although it may not be an independent prognostic factor when adjusted for molecular classification [26, 27] (Fig. 3).

### Ovarian cancer: a low-frequency event with subtype specificity and unique significance

In contrast to endometrial cancer, *PPP2R1A* mutations are generally rare in ovarian cancer. Their frequency also exhibits histologic subtype dependence: they are extremely rare in high-grade serous ovarian cancer (HGSOC) (0–4.5%) but are relatively more common in Type I ovarian cancers, though overall frequencies remain low [28]. For instance, the frequency is 4.5–9.1% in ovarian clear cell carcinoma (OCCC), 10% in low-grade endometrioid carcinoma, and has conflicting reports in mucinous carcinoma (one study reported 33.3%, another 0%) [28, 29].

Notably, the mutational hotspot in ovarian cancer is distinct from that in endometrial cancer, primarily affecting the R183 codon. This differential preference for hotspots between gynecologic cancers provides a critical clue for understanding their tissue-specific oncogenic mechanisms [30]. Furthermore, in OCCC, *PPP2R1A* mutations often co-occur with *ARID1A* mutations, suggesting a synergistic role for these two pathways in OCCC pathogenesis [31].

### Cervical cancer and other gynecologic malignancies: a rare event

In cervical cancer, *PPP2R1A* mutations are exceedingly rare, with a frequency of only 0–1% [32]. Despite this, *PPP2R1A* mutation status is used as an inclusion criterion in several clinical trials for cervical cancer, indicating that researchers believe targeting this pathway may hold therapeutic potential [33]. The cBioPortal database also documents a case of a cervical squamous cell carcinoma patient with a *PPP2R1A* P179L mutation. *PPP2R1A* mutations have also been observed in other gynecologic malignancies, such as vulvar cancer [22].

The distinct mutational hotspot patterns of *PPP2R1A* in different gynecologic tumors (P179/S256 in endometrial cancer vs. R183 in ovarian cancer) are not random genetic drift but rather hint at a profound, tissue-specific functional context [26]. A plausible explanation for this phenomenon is that cells from different tissues possess unique protein interaction networks, or “interactomes [34].” Endometrial and ovarian cells likely express different repertoires of B subunits, endogenous inhibitors, and other interacting proteins. Consequently, the mutations that most effectively disrupt the specific PP2A-B subunit complexes critical for tumorigenesis in a given tissue environment will gain the strongest selective advantage [35]. For example, the P179R mutation may be most effective at relieving inhibition of a specific oncogenic pathway in the endometrial context, while the R183W mutation achieves a similar effect in the ovarian environment [36].

Recent structural biology and proteomic studies support this framework, demonstrating that specific *PPP2R1A* mutants exhibit differential binding affinities for various B-subunits. These variations in holoenzyme assembly dictate the specific downstream signaling pathways altered in endometrial versus ovarian tissues [37]. This reveals a highly refined, cell-context-dependent oncogenic mechanism and provides a rationale for developing tissue-specific targeted therapies (Table 1).

**Table 1:** Frequency and characteristics of *PPP2R1A* mutations in major gynecologic malignancies

Cancer Type	Histologic Subtype	Mutation Frequency (%)	Common Hotspot Codons	Key Co-mutations	Key References
Endometrial Cancer	Type II (Serous, Carcinosarcoma)	Up to 40%	P179, S256	*TP53*	[38]
	Type I (Endometrioid)	0–7%	None specific	PTEN, PIK3CA	[39]
Ovarian Cancer	Clear Cell (OCCC)	4.5–9.1%	R183	*ARID1A*, PIK3CA	[40]
	High-Grade Serous (HGSOC)	0–4.5%	R183	*TP53*	[41]
	Low-Grade Endometrioid	10%	R183	PIK3CA	[42]
Cervical Cancer	Squamous Cell, Adenocarcinoma	0–1%	P179L (case report)	PIK3CA, KRAS	[43]

## The immunomodulatory role of *PPP2R1A* mutations: shaping an immunologically “Hot” tumor microenvironment

### A new predictive biomarker: *PPP2R1A* mutations portend exceptional survival benefit from immune checkpoint inhibitor therapy

In recent years, a pivotal study published in Nature, led by Jazaeri et al., has significantly advanced our understanding of *PPP2R1A* mutations. The study focused on a cohort of 34 patients with refractory ovarian clear cell carcinoma (OCCC) treated with a combination of durvalumab (a PD-L1 inhibitor) and tremelimumab (a CTLA-4 inhibitor). The clinical data revealed clinically significant results: patients with *PPP2R1A* mutations had a median overall survival (OS) of 66.9 months, compared to just 9.2 months for patients with wild-type *PPP2R1A* [33]. The universality of this finding was subsequently validated in external cohorts, including an endometrial cancer cohort and a pan-cancer cohort of over 9,000 patients treated with ICI, both of which confirmed the association between *PPP2R1A* mutations and survival benefit following ICI therapy [33]. Importantly, in OCCC, the predictive power of *PPP2R1A* mutation status for survival surpassed that of *ARID1A* mutations, which had previously garnered significant attention [33].

### Reshaping the tumor immune microenvironment (TME): analysis of key immunophenotypic changes

The remarkable survival advantage conferred by *PPP2R1A* mutations is rooted in their ability to transform the tumor immune microenvironment (TME) from a “cold” (immunosuppressive) state to a “hot” (immuno-activated) state.

#### Upregulation of the interferon-γ (IFNγ) signaling axis

Translational analysis of patient tumor biopsies revealed that, both at baseline and after ICI treatment, *PPP2R1A*-mutant tumors exhibited significantly enhanced IFNγ signaling pathway activity [44]. IFNγ signaling is a classic hallmark of an activated anti-tumor immune response, and its upregulation signifies active immune surveillance and attack within the TME [45].

#### Formation of tertiary lymphoid structures (TLS)

Researchers observed the presence of tertiary lymphoid structures (TLS) in baseline samples of *PPP2R1A*-mutant tumors. TLS are ectopic lymphoid organs that form in non-lymphoid tissues, such as tumors, during chronic inflammation. Functionally analogous to lymph nodes, they can initiate and sustain powerful adaptive immune responses in situ [46]. The presence of TLS is considered a strong predictor of a favorable response to cancer immunotherapy.

#### Enhanced infiltration and expansion of effector T cells (CD8+)

The most direct evidence comes from observations of T cells. Analysis showed that following ICI treatment, the microenvironment of *PPP2R1A*-mutant tumors displayed significantly enhanced immune cell infiltration, particularly a substantial expansion in the number of CD45RO+CD8 + T cells (memory effector T cells) [33]. This indicates that a robust anti-tumor attack, led by the adaptive immune system, is occurring within the tumor. This finding is consistent with the general conclusion that tumor-infiltrating lymphocytes (TILs) have prognostic value in ovarian cancer [47].

### The mechanistic basis of immune activation

A single genetic event, a *PPP2R1A* mutation, can initiate a unified cascade that connects multiple pillars of cancer immunology. It not only creates the “fuel” for an immune attack (neoantigens) but also simultaneously activates the “alarm system” of innate immunity (cGAS-STING) and ultimately recruits the “army” of the adaptive immune system (T cells) [48, 49]. This synergistic effect explains its potent ability as a predictive biomarker.

#### The intersection with the DNA damage response (DDR): fostering genomic instability and neoantigen production

PP2A plays a significant role in the DNA damage response (DDR). When PP2A function is inhibited, the cell’s DNA repair mechanisms are compromised. Therefore, loss-of-function mutations in *PPP2R1A* directly weaken PP2A’s role in the DDR, leading to increased genomic instability [50]. The downstream consequence of this process is critical: persistent genomic instability and DDR defects cause the tumor cells to accumulate mutations during division, thereby generating a large number of tumor-specific neoantigens. These neoantigens act as foreign “tags” that can be recognized by the immune system, exposing the previously “invisible” tumor cells to surveillance by CD8 + T cells [51].

#### Unleashing innate immunity: The role of the cGAS-STING pathway

This mechanism is the key bridge connecting genomic instability to adaptive immunity. The genomic instability caused by PP2A/DDR dysfunction leads to the formation of micronuclei and chromosomal fragments, from which DNA can leak into the cytoplasm, forming free double-stranded DNA (dsDNA) [52]. This aberrant cytosolic dsDNA is recognized by a sensor called cGAS, which in turn activates the STING signaling pathway [53]. STING activation triggers a potent innate immune response, the core of which is the production of large amounts of Type I interferons (IFNs) [54]. Type I IFNs are critical messengers of the immune system; they promote the maturation of dendritic cells (DCs) and the cross-presentation of antigens, effectively transmitting the signal from the innate immune response to the adaptive immune system, thereby initiating and amplifying the CD8 + T cell response against tumor neoantigens [55]. Furthermore, research has found that PP2A itself negatively regulates the STING pathway in macrophages via the Hippo-YAP/TAZ axis, meaning that loss of PP2A function may also relieve STING inhibition in immune cells, further amplifying the overall immune activation effect [56].

#### The Wnt signaling paradox: coordinating pro-metastatic and pro-immune functions

The research presents seemingly contradictory evidence: some data suggest that *PPP2R1A* mutations may promote tumor metastasis by enhancing Wnt signaling and the secretion of Wnt ligands, while Wnt signaling is known in some contexts to create an immunosuppressive TME [57, 58]. This paradox can be reconciled by considering the state of the TME. In an immunologically “cold” environment, enhanced Wnt signaling may indeed promote metastasis. However, in the context of the strong immune activation driven by the DDR/STING axis initiated by *PPP2R1A* mutations, this pro-immune effect clearly predominates. The theoretical framework for this dominance lies in the massive influx of highly immunogenic neoantigens combined with STING-mediated type I interferon production, which profoundly reshapes the local chemokine milieu. This acute, high-intensity inflammatory signaling overrides the baseline immunosuppressive signals typically maintained by Wnt, shifting the balance from immune exclusion to robust immune infiltration [59, 60]. When immune checkpoint inhibitors further release the brakes on the immune system, this effect is magnified, and the resulting survival benefit is sufficient to override any potential negative impact from Wnt signaling [61]. Therefore, the immune status of the TME is the critical variable determining the ultimate outcome of Wnt pathway activation.

A key observation in endometrial cancer is that *PPP2R1A* mutation status is not associated with PD-L1 expression levels. This indicates that *PPP2R1A* mutations do not simply enhance ICI efficacy by increasing the PD-L1 target but rather serve as a marker of the tumor’s “intrinsic immunogenicity [56].” ICI is effective because the TME is already inflamed by the STING/IFNγ axis and filled with primed T cells. In this highly inflammatory environment, even baseline levels of PD-1/PD-L1 interaction constitute a major restraint on the immune system [61]. Therefore, releasing this “brake” produces a far more significant effect than it would in an immunologically “cold” environment. This makes *PPP2R1A* mutation status a more fundamental and reliable predictive biomarker for immunotherapy response than PD-L1 expression itself (Table 2).

**Table 2:** Immunophenotype and signaling pathway changes in *PPP2R1A*-mutant tumors

Molecular Event	Key Signaling Pathway	Consequence in Tumor Cells	Consequence in TME	Key References
*PPP2R1A* Mutation	PP2A Loss-of-Function	Dysregulation of oncogenic pathways (e.g., AKT, MAPK)	-	[19, 25]
Loss of Phosphatase Activity	DNA Damage Response (DDR)	Impaired DNA repair, increased genomic instability	-	[62]
Genomic Instability	-	Neoantigen production, cytosolic dsDNA accumulation	-	[63]
Cytosolic dsDNA	cGAS-STING Pathway	Innate immune signaling activation	-	[64]
STING Activation	Type I Interferon (IFN) Signaling	-	Production of large amounts of Type I IFNs, promotion of DC maturation	[65]
Type I IFN Upregulation	IFNγ Signaling	-	Enhanced IFNγ signaling, promotion of T cell cross-presentation	[66]
Adaptive Immune Activation	-	-	CD8 + T cell infiltration and expansion, TLS formation	[67]

PP2A Loss-of-Function: loss of phosphatase activity and the resulting dysregulation of downstream oncogenic pathways

## Therapeutic strategies targeting the *PPP2R1A*/PP2A pathway

The biological characteristics revealed by *PPP2R1A* mutations have paved the way for two distinct but logically consistent therapeutic strategies: first, exploiting the genetic vulnerability they create for precision targeting, and second, using drugs to mimic their loss-of-function to sensitize tumors to immunotherapy.

### Exploiting genetic vulnerability: synthetic lethality with DNA damage response inhibitors

The core idea of a synthetic lethality strategy is that *PPP2R1A*-mutant tumor cells, due to their inherent DDR defects, will be highly sensitive to drugs that target other DDR pathways.

ATR Inhibitors (ATRi): Preclinical studies have confirmed that in OCCC, *PPP2R1A* mutations significantly enhance cellular sensitivity to ATRi, even in cells that already have *ARID1A* defects. The application of ATRi led to more severe genomic instability and apoptosis [30].

RNR Inhibitors: Similarly, in *PPP2R1A*-mutant uterine serous carcinoma cells, ribonucleotide reductase (RNR) inhibitors have shown a synthetic lethal effect [68].

Clinical Translation: This strategy has entered the clinical validation phase. In patients with *PPP2R1A*-mutant ovarian and endometrial cancer, the combination of the PKMYT1 inhibitor lunresertib with the ATRi camonsertib has shown encouraging early clinical data [69].

### The duality of PP2A regulation: a tale of two therapeutic approaches

Drug development targeting PP2A presents an interesting duality, with both PP2A activators and inhibitors being pursued. This may seem contradictory, but the therapeutic logic depends on the underlying cause of PP2A dysfunction in a specific tumor.

#### PP2A reactivation for direct anti-tumor effects

Rationale: This strategy is suitable for tumors where the *PPP2R1A* gene is wild-type, but PP2A function is suppressed by other mechanisms, such as the overexpression of endogenous inhibitors (e.g., CIP2A, SET) [70, 71].

Mechanism of Action: PP2A small-molecule activators (SMAPs), which work by binding to the A-C subunit interface to promote holoenzyme assembly, or compounds like FTY720 (fingolimod, an FDA-approved sphingosine 1-phosphate receptor modulator that indirectly activates PP2A) [72], can restore the tumor-suppressive activity of PP2A, thereby inhibiting cancer cell growth [73].

Preclinical Evidence: In USC cells with the P179R mutation, introducing wild-type Aα or using a SMAP was able to inhibit tumor growth, suggesting that even in a mutant background, increasing overall PP2A activity remains effective. Furthermore, SMAPs have shown efficacy in various other cancer models, such as prostate cancer and neuroblastoma [74, 75].

#### PP2A inhibition to enhance immunotherapy

Rationale: This strategy aims to pharmacologically mimic (phenocopy) the pro-immune effects of *PPP2R1A* loss-of-function mutations in *PPP2R1A* wild-type tumors. The core objective is to convert immunologically “cold” tumors into “hot” ones [76].

Mechanism of Action: PP2A inhibitors (e.g., LB-100) suppress PP2A activity, thereby inducing genomic instability, neoantigen production, and activation of the cGAS-STING pathway. This enhances the T cell response and sensitizes the tumor to ICI therapy [77].

Preclinical Evidence: LB-100 has demonstrated strong synergistic effects with PD-1 inhibitors in various tumor models (e.g., glioblastoma, colorectal cancer, pancreatic cancer, triple-negative breast cancer), effectively converting “cold” tumors into “hot” ones [78]. However, it is important to note that LB-100 can also inhibit other protein phosphatases, such as PP1 and PP5. While the net effect in preclinical models is a robust pro-immune conversion of the TME, the exact extent to which the off-target inhibition of PP1 or PP5 contributes to or hinders this ICI synergy remains an open question requiring further mechanistic investigation [79].

### Clinical development and combination therapies in gynecologic cancers

Strategies targeting the PP2A pathway are actively being translated into the clinic. A clinical trial led by Dr. Jazaeri at MD Anderson Cancer Center is evaluating the efficacy of the PP2A inhibitor LB-100 in combination with an immune checkpoint inhibitor for the treatment of OCCC, a direct clinical validation of the findings from the Nature study. Additionally, multiple clinical trials have included *PPP2R1A* mutation status as an enrollment criterion, involving drugs such as ATRi (lunresertib + camonsertib) in therapeutic regimens for various solid tumors, including ovarian, endometrial, and cervical cancers (Table 3) [33].

**Table 3:** Selected clinical trials involving *PPP2R1A* status or PP2A-targeting drugs in gynecologic cancers

Trial Identifier (NCT#)	Drug(s)/Therapy	Mechanism of Action	Combination Partner	Cancer Type(s)	Phase	Key Biomarker	Key References
NCT03026062 (Related Study)	Durvalumab + Tremelimumab	PD-L1 inhibitor + CTLA-4 inhibitor	-	Ovarian Clear Cell Carcinoma	II	*PPP2R1A* mutation	[80]
LIXTE OCCC Trial	LB-100 + Dostarlimab	PP2A inhibitor + PD-1 inhibitor	Dostarlimab	Ovarian Clear Cell Carcinoma	II	*PPP2R1A* status	[81]
Not specified	Lunresertib + Camonsertib	PKMYT1 inhibitor + ATR inhibitor	Camonsertib	Ovarian Cancer, Endometrial Cancer	I/II	*PPP2R1A* mutation	[69]
Multiple Trials	Various drugs	-	-	Cervical Cancer	I, I/II	*PPP2R1A* status	[22]
Multiple Trials	Various drugs	-	-	Ovarian Cancer	I, I/II	*PPP2R1A* status	[30]

## Synthesis, unanswered questions, and future perspectives

### Synthesis: reconciling the paradox of a lost tumor suppressor as a favorable immunotherapy biomarker

**Fig. 4:**
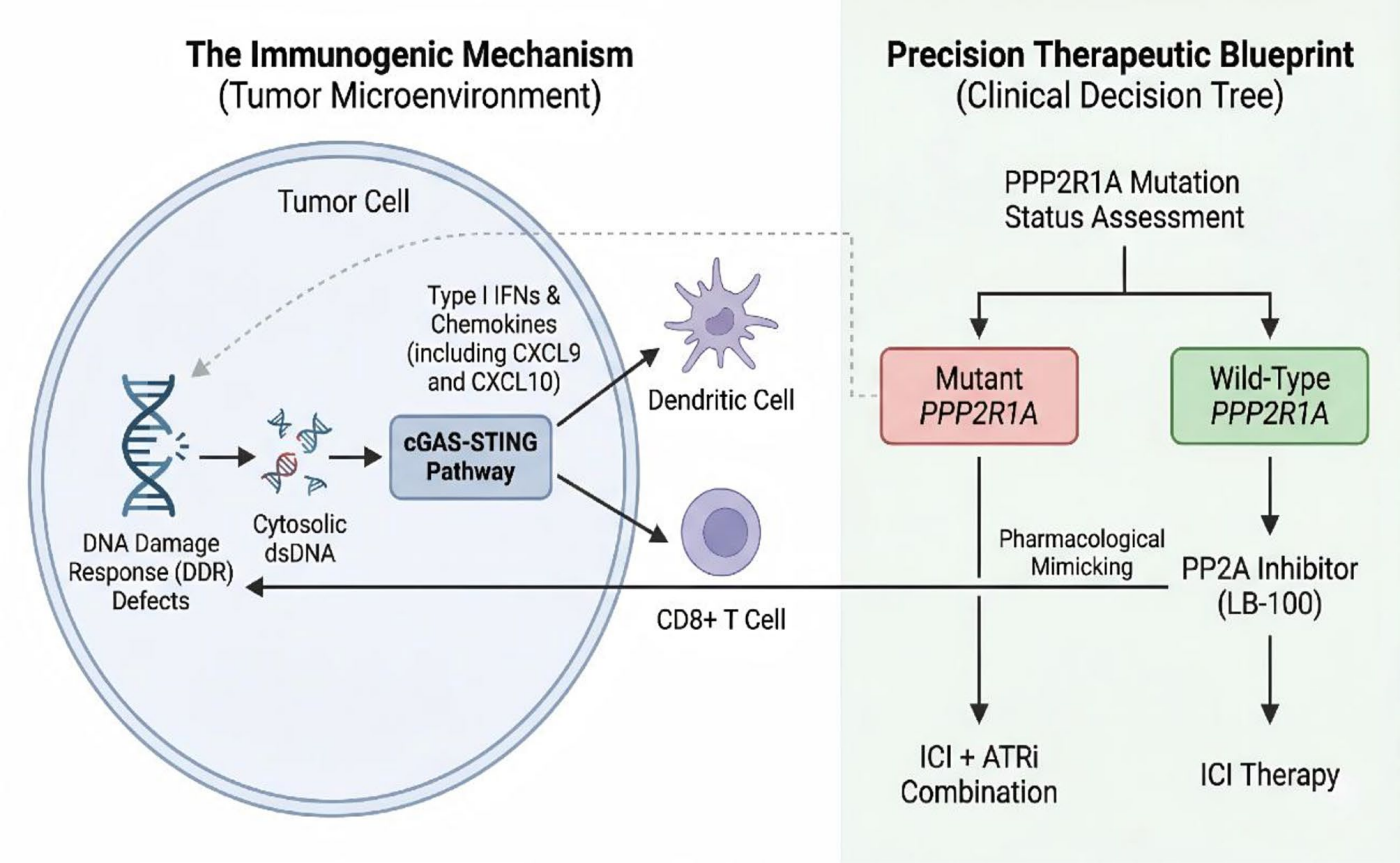
Integrated schematic of the cGAS-STING immunogenic cascade and precision therapeutic blueprint. The left panel delineates the mechanism underlying the “cold” to “hot” tumor microenvironment transition. PP2A dysfunction leads to DNA damage response (DDR) defects and the subsequent accumulation of cytosolic dsDNA. This directly activates the cGAS-STING pathway, driving the secretion of Type I interferons and specific chemokines (CXCL9 and CXCL10) to recruit dendritic cells and CD8 + T cells. The right panel outlines a stratified clinical decision tree based on PPP2R1A mutation status. Mutant PPP2R1A tumors are targeted with an ICI and ATRi combination to exploit synthetic lethality. Conversely, wild-type PPP2R1A tumors are treated with a PP2A inhibitor (LB-100) to pharmacologically mimic the mutated phenotype, thereby sensitizing the tumor to ICI therapy

The study of *PPP2R1A* has revealed and ultimately resolved a profound biological paradox. The oncogenic loss-of-function of *PPP2R1A* is a double-edged sword: on one hand, it drives malignant transformation by relieving the brakes on growth pathways like AKT and MAPK [19]; on the other hand, by weakening the DNA damage response system, it embeds a deep genetic vulnerability within the tumor [68]. It is precisely this vulnerability that unleashes a powerful, self-sustaining immunogenic cascade (genomic instability → neoantigens → STING → interferons → T cells), rendering the tumor cells highly susceptible to immune-mediated killing, especially when combined with immune checkpoint inhibitors (Fig. 4).

### Filling the gaps: unanswered questions and potential resistance mechanisms

Despite significant progress, many critical questions remain unanswered, and vigilance against potential resistance mechanisms is required.

#### Unanswered Questions

Tissue Specificity of Hotspot Mutations: Why do endometrial and ovarian cancers select for different *PPP2R1A* hotspot mutations (P179/S256 in EC vs. R183 in OCCC)? What are the underlying molecular mechanisms? (Derived from Sect.  2 analysis).

Full Spectrum of B Subunit Analysis: While initial immunoprecipitation-mass spectrometry (IP-MS) experiments have begun to map how specific mutant proteins alter B subunit binding profiles, a comprehensive interactome landscape across all gynecologic tissue contexts is still lacking. Furthermore, exactly how these altered binding affinities directly translate into tissue-specific oncogenesis requires deeper exploration [15, 30, 37].

Duality of Wnt Signaling: Is it therapeutically possible to separate the pro-metastatic effects of Wnt signaling from its pro-immune effects?

#### Potential Resistance Mechanisms

Although there are no studies specifically on ICI resistance mechanisms in *PPP2R1A*-mutant tumors, general principles of immunotherapy suggest the following possibilities:

Tumor-Intrinsic Factors: In a high-neoantigen context, tumor cells frequently evade immune surveillance through profound antigen loss or the downregulation of human leukocyte antigen (HLA) class I molecules, rendering the mutated peptides invisible to T cells. Additionally, tumors may acquire secondary mutations in key molecules of the IFNγ signaling pathway (e.g., JAK/STAT) or upregulate alternative immune checkpoints (e.g., TIM-3, LAG-3).

Tumor-Extrinsic Factors: T cells within the TME may become exhausted due to chronic antigen stimulation; the recruitment of other immunosuppressive cells (e.g., myeloid-derived suppressor cells, MDSCs) could reverse the immune-activated state, or TLS may fail to maintain their function.

### Conclusion and future outlook: integrating *PPP2R1A* into the precision immuno-oncology framework

Research into *PPP2R1A* has opened a new chapter in the treatment of gynecologic cancers, with broad clinical and therapeutic prospects.

Clinical Practice Implications: It is strongly recommended to incorporate *PPP2R1A* mutation testing into the standard molecular profiling of gynecologic malignancies, particularly OCCC and USC, to accurately identify the patient population that will derive the greatest survival benefit from immunotherapy.

Future Therapeutic Directions: Based on current research, a two-pronged future therapeutic blueprint can be envisioned:

For *PPP2R1A*-mutant tumors: Utilize immune checkpoint inhibitors as the core therapy and explore combinations with DDR inhibitors (such as ATRi) to potentially deepen and prolong the treatment response.

For *PPP2R1A* wild-type tumors: Employ a combination strategy of PP2A inhibitors (like LB-100) with immune checkpoint inhibitors. This approach aims to pharmacologically mimic the immunogenic effects of the mutation, converting immunologically “cold” tumors into “hot” ones, thereby expanding the population of patients who can benefit from immunotherapy.

In conclusion, the in-depth study of *PPP2R1A* has not only unveiled the intrinsic links among a core tumor suppressor, genomic integrity, and anti-tumor immunity but has also opened a new and hopeful era for tackling the significant challenge of aggressive gynecologic malignancies.

## Data Availability

Not applicable.

## Abbreviations

PP2A: Protein Phosphatase 2 A
ICI: Immune Checkpoint Inhibitor
USC: Uterine Serous Carcinoma
UCS: Uterine Carcinosarcoma
EC: Endometrial Cancer
EEC: Endometrioid Endometrial Cancer
HGSOC: High-Grade Serous Ovarian Cancer
OCCC: Ovarian Clear Cell Carcinoma
TME: Tumor Microenvironment
IFNγ: Interferon Gamma
TLS: Tertiary Lymphoid Structures
TILs: Tumor-Infiltrating Lymphocytes
DDR: DNA Damage Response
dsDNA: Double-Stranded DNA
DCs: Dendritic Cells
IFN: Interferon
OS: Overall Survival
SMAPs: Small-Molecule Activators of PP2A
ATRi: ATR Inhibitors
RNR: Ribonucleotide Reductase
MDSCs: Myeloid-Derived Suppressor Cells
